# Long-term whiplash-associated disorders, sickness absence, and disability pension following rear-end car crashes and associations with whiplash protection systems: a longitudinal cohort study

**DOI:** 10.1186/s12889-026-27404-2

**Published:** 2026-04-20

**Authors:** Rasmus Elrud, Emilie Friberg, Alejandra Machado, Kristina Alexanderson, Helena Stigson

**Affiliations:** 1 Division of Insurance Medicine, Department of Clinical Neuroscience, Karolinska Institutet, Stockholm, SE-171 77 Sweden; 2 Folksam Research, Folksam Insurance Group, Stockholm, Sweden

**Keywords:** Traffic accidents, WAD, Whiplash, Sick leave, Disability pension, Impairment

## Abstract

1. Older cars without WPS were linked to higher long-term WAD and PMI, especially in men.

2. WPS offer more protection to men than women in rear-end collisions.

3. Long-term WAD leads to sustained increases in sickness absence and disability days.

4. Newer cars without WPS still show increased risk for men.

5. Improved WPS design is needed to better protect women in crashes.

**Supplementary Information:**

The online version contains supplementary material available at 10.1186/s12889-026-27404-2.

## Abstract (structured)

**Background:**

Whiplash-associated disorders (WAD) are common injuries from car crashes, with a significant proportion leading to permanent medical impairment (PMI), especially among women and in rear-end impacts. While previous research has shown mixed results regarding work incapacity following WAD, severe cases may lead to long-term health and functional consequences. This study aimed to examine the occurrence of long-term WAD – based on insurance compensation, future PMI, and work incapacity (sickness absence (SA) and disability pension (DP)) among front seat occupants in rear-end crashes, and to assess how seat design with or without whiplash protection systems (WPS) relates to these outcomes.

**Methods:**

The cohort (*N* = 14,363) was selected from car crashes occurring 2001–2013, reported to the Folksam Insurance Group. Injury- and car-related information was linked with nationwide register data regarding sociodemographics, inpatient and specialized outpatient healthcare, SA, and DP. The SA + DP days/year were calculated in relation to the crash date. Logistic regression analyses were performed with variables combining WPS and car model year of introduction (< 1998 and ≥ 1998) for long-term WAD, PMI, and > 90 days of all diagnoses and whiplash-specific SA/DP in year two after the crash. Analyses were stratified by sex.

**Results:**

Among women, 18% sustained long-term WAD and 12% resulted in PMI. Among men,13% and 9%, respectively. Among occupants with long-term WAD, there was a substantial increase in mean SA/DP days/year, WAD specific and all diagnoses, after the crash and remained high three years after. Compared to occupants in cars with WPS, occupants in older cars (< 1998) without WPS were more likely to have long-term WAD (Women: OR:1.8, 95% confidence intervals (CI):1.5–2.2; Men: OR:2.2, 95% CI:1.7–2.8), and PMI (Women: OR:1.4, 95% CI:1.1–1.8; Men: OR:2.3, 95% CI:1.6–3.3). Among men, also occupants in newer cars without WPS were more likely to have long-term WAD (OR:1.6; 95% CI:1.2-2.0) and PMI (OR:1.9; 95% CI;1.3–2.7). Among men, but not women, those injured in older cars without WPS were more likely to have > 90 days SA/DP in year two following the crash (OR:1.8; 95% CI: 1.2–2.6).

**Conclusion:**

WPS were associated with a lower risk of long-term WAD, PMI, and prolonged SA/DP, particularly among men, with substantial less benefit for women. These sex differences highlight the need to improve WPS performance, especially for women.

**Supplementary Information:**

The online version contains supplementary material available at 10.1186/s12889-026-27404-2.

## Introduction

Whiplash-associated disorders (WAD) constitute a substantial proportion of all injuries from car crashes [1], and more than 50% of all injuries result in permanent medical impairment (PMI) [2]. 44% of all WAD resulting in PMI, occurred in rear impacts [3]. This distribution reflects exposure patterns, as the risk of PMI given a WAD is the same regardless of impact direction [4]. Both the injury risk and the risk of long-term consequences in terms of WAD in terms of PMI are higher among women than among men [5]. One study indicated a significant and lasting decline in physical quality of life, still five years after the accident, among individuals with severe WAD compared to individuals with other mild injuries [6].

Work incapacity after a crash has been studied in relation to WAD, with great variety in measures and study populations, and results go in different directions. Some studies have reported high proportions returning to work with short time off from work [7, 8], while others have reported high proportions still on sickness absence (SA) or disability pension (DP) several years after the crash [9, 10].

Interpretations of results and comparison between studies is difficult, due to that, e.g., many of the studies suffer from small sample sizes, large drop-outs, and using measures of WAD and work incapacity that are not certified by a physician, e.g., self-reported data. Also, many studies examining SA and DP after WAD have relatively short follow-up periods.

Long-term WAD and SA/DP among injured occupants with WAD and with any type of injuries have been reported by previous studies [11–13]. These findings highlight the importance of including previous SA when investigating SA/DP following a crash.

It has been shown that the seat design is the most influential factor to reduce WAD in rear-end crashes [14]. Since the late 1990s many car manufacturers have introduced different measures to prevent WAD in rear-end crashes [15–17]. Some approaches to reduce the risk are minimizing the relative motion between head and torso, controlling the energy transfer between the seat and the human body, and/or absorbing energy in the seat back [18]. These systems vary regarding how effective they are in reducing the risk of WAD [3, 19] and the risk that the WAD results in PMI [3]. Furthermore, the risk reduction of these systems have been found to be less effective for women compared to men, with some systems even performing worse than standard seats without such systems [3]. No study has, to the best of our knowledge, examined the association between whiplash protection systems (WPS) and SA and DP after a car crash.

Therefore, we aimed to explore the occurrence of long-term WAD, future PMI, and SA/DP (SA/DP for all diagnoses and SA/DP specifically related to WAD diagnoses) among front seat car occupants with WAD injured in rear-end crashes, accounting for sociodemographics factors, with a particular focus on sex differences. Another aim was to study the associations between WPS and long-term WAD, and future PMI and SA/DP.

## Materials and methods

A longitudinal register-based cohort study was conducted, comprising individuals injured in a car crash in the years 2001–2013, reported to Folksam Insurance Group (Folksam), one of Sweden’s largest insurance companies. Folksam has an approximately 20% market share of the mandatory road traffic insurance in Sweden. Inclusion criteria were: reporting WAD as a front seat occupant involved in a rear-end crash when aged 17–62 (i.e., those that were at risk of DP during the follow-up regarding age (19–64)).

Information regarding the crash (crash date, position in car, crash impact direction, initial injuries, PMI, compensations for injury, car model year of introduction (MYI) and whiplash protection system of the car) was obtained from Folksam.

Further information for each individual was obtained from four nationwide registers, through linkage at individual level, using the personal identity number assigned to all residents of Sweden and obtained from the following three authorities:

- The National Board of Health and Welfare: Dates and injury diagnoses from inpatient and specialized outpatient healthcare, coded according to the International Classification of Diseases 10th revision (ICD-10) in the *National Patient Register (NPR)*; and death dates from the *Cause of Death Register*.
- Statistics Sweden: Sociodemographics from December the year before the crash and emigration year from the *Longitudinal Integration Database for Health Insurance and Labour Market Studies (LISA)*.
- Social Security Agency: Dates and diagnoses of SA spells > 14 days and of DP for the studied years from the *MicroData for Analysis of the Social Insurance database (MiDAS)*.

Individuals were excluded if dead within 30 days after the crash date (T_0_; *n* = 0) or had another crash within three years following T_0_ (*n* = 96; 0,66%). This resulted in a cohort of 14,363 individuals.

Information regarding WAD was obtained from Folksam or in case of no such information, from NPR (ICD-10 code S134), registered at healthcare visits up to four days after T_0_. Four days was chosen based on distribution of healthcare with such injuries around T_0_.

### Seat design

To interpret the associations of seat design with whiplash injury outcomes, it is essential to distinguish between the different whiplash protection concepts that have been introduced over time. Since 1998, several systems have been developed, including proactive, reactive, and passive designs. These concepts vary in how they interact with the occupant during a rear‑end impact, either by activating head restraints (Pro‑Active Head Restraints, PAHR), allowing the seatback to move in a controlled way (e.g., WHIPS, RHR, SAHR) or by using optimized passive designs without moving components (PAS, PAS_TO) [15–17].

In the present study, the seat designs were categorized in two ways. The first categorization was made according to all seat concepts manufactured in 1998 or after (MYI ≥ 1998) (see Table 1), and cars manufactured before 1998, before this concept was implemented (MYI < 1998).

**Table 1:** Summary of seat design concepts and analytical categories

Seat Concept	Description	Examples / Manufacturers	Analytical Category
PAHR (Pro-Active Head Restraints)	Sensors activate head restraint early or pre-crash to reduce head-to-restraint distance.	Various manufacturers	WPS, MYI ≥ 1998
PAS (Passive Seats)	Energy-absorbing structure and optimized geometry; no active components.	Audi, Volkswagen, Ford	WPS, MYI ≥ 1998
PAS_TO (Toyota Passive Seat Concept)	Toyota’s energy-absorbing passive seat concept.	Toyota	WPS, MYI ≥ 1998
RHR (Reactive Head Restraints)	Head restraint moves forward when the torso loads the seatback.	Audi, Mercedes, Nissan, Opel, Volkswagen	WPS, MYI ≥ 1998
RHR_SAHR (Saab Active Head Restraints)	Saab-specific reactive head restraint introduced in 1998.	Saab	WPS, MYI ≥ 1998
WHIPS (Volvo Whiplash Protection System)	Reactive seatback design that moves backward/forward to reduce neck loads.	Volvo	WPS, MYI ≥ 1998
STD (Standard Seats without any advanced whiplash concept)	Standard seats without advanced whiplash concepts in newer vehicles.	Various	No system, MYI ≥ 1998
Cars < 1998	Older seats without WPS; concepts not yet introduced.	All manufacturers	No system available, MYI < 1998

*Abbreviations*: *WPS* Whiplash protection system, *MYI* Model year of introduction

The second categorization groups together cars into three categories: those manufactured since 1998 with WPS (WPS MYI ≥ 1998), those manufactured since 1998 without such systems (No system MYI ≥ 1998), and older cars manufactured before 1998, which did not have any protection systems available (MYI < 1998, no system available).

### Outcomes

Long-term WAD was defined as having received insurance compensation from Folksam (corresponding to four weeks or more, ≥ 2000 SEK/190 EUR), with eligibility determined through medical certification or documentation of related healthcare expenses.

In Sweden, traffic injuries are compensated by the Swedish Road Traffic Insurance [20]. If an occupant has not recovered within two years, a physician with a board certified speciality asses if the injury and the symptoms can be traced to the crash, based on medical records from before and after the crash [21]. If so, the reduction in function is graded between 0 and 99%, based on principles for grading of medical impairment degree, which were established in the early 20th century in consensus between physicians, and applied by all Swedish insurance companies. Here the outcome used was: no PMI (WAD did not result in PMI) or PMI (WAD resulting in ≥ 1% PMI). Thus, the category ‘PMI’ here denotes the threshold at which an individual was considered to have any degree of PMI.

In Sweden, all residents with income from work or unemployment benefits can be granted SA benefits through the public SA insurance, if a disease or injury has led to work incapacity [22]. Generally, the first 14 days of a SA spell is paid by the employer, thereafter by the Social Insurance Agency. Therefore, we included the SA spells > 14 days. In case of permanent work incapacity due to disease or injury, all residents aged 19–64 years could be granted DP. Up to a certain level, SA benefits amount to 80% and DP to 64% of lost income. Both SA and DP can be granted for full- or part-time of ordinary working hour, thus, people can have part-time SA and DP at the same time. Therefore, we calculated net days, e.g., two gross days of 50% equals one net day. From here on, SA and DP days always refer to net days. The annual number of combined SA and/or DP (SA/DP) days two years before and three years after T_0_ (Y_− 2_ to Y_+ 3_) was calculated for each individual. Two outcomes were used: Annual days of SA/DP irrespective of diagnoses (hereafter, all-cause SA/DP), and annual days of SA/DP due to WAD diagnosis. SA and DP diagnoses are coded according to the ICD-10, on the 3-digit level. Thus, S13, “Dislocation, sprain and strain of joints and ligaments at neck level”, was used to identify SA and DP with WAD diagnosis. In some analyses SA/DP was categorized into ≤ 90 and > 90 days. A second categorization of SA/DP with WAD diagnosis, using cut-off ≤ 30 days and > 30 days was also tested.

### Covariates

Information on sociodemographic covariates from the year prior to the car crash (Y_− 1_): sex (women, men), age (17–29, 30–44, 45–59, 60–62 years), educational level (elementary school (≤ 9 years or missing information), high school (10–12 years), college/university (> 12 years)), country of birth (Sweden, rest of Europe, rest of the world (including missing information)), and married (yes/no).

Two covariates regarding SA/DP prior to T_0_: Previous SA, defined as > 90 days during Y_− 1_ (yes/no), and already ongoing DP at T_0_ (yes/no).

Two covariates regarding safety level of the car: MYI and car size. When not used in the exposure variable combining seat design and MYI, MYI was categorized: ≤1993 (including cars with missing values), 1994–1998, 1999–2003, 2004–2008, 2009–2013. Car size was categorized as: supermini, small family car, large family car, executive car, minibus, SUV, missing information.

To take into consideration possible changes over time in the process of determining PMI and granting SA and DP, the year of the crash was taken into consideration, categorized as: 2001–2004, 2005–2008, 2009–2013.

### Statistical analyses

Because of the well-known differences between women and men regarding risk of WAD and WAD resulting in PMI [3], all analyses were stratified by sex. Descriptive statistics were calculated. Mean number of SA/DP days/year during Y_− 2_-Y_+ 3_ was calculated, with individuals not at risk of SA and DP (due to migration or death) excluded for each year.

Logistic regression analyses were used to study associations between seat design and the different outcomes by estimating odds ratios (OR) with 95% confidence intervals (CI). Multivariate models were adjusted for potential confounders based on previous literature. Models using long-term WAD or PMI as outcome included all 14,363 individuals in the cohort. Models with outcome > 90 days SA/DP in Y_+ 2_, excluded individuals not at risk of SA/DP in Y_+ 2_ due to already ongoing DP at T_0_ (*n* = 1241), migration (*n* = 10), or death (*n* = 23), leaving 13,089 (women: 6519, men: 6570).

A low number of occupants in cars with WPS with > 90 days SA/DP with WAD diagnoses in Y_+ 2_ did not allow for analyses with separate categories for different systems for SA/DP with WAD specific diagnoses (see Tables in Supplementary material). The lower cut-off, ≤ 30 days and > 30 days, did still not allow detailed analysis.

For statistical analyses, SAS 9.4 and IBM SPSS Statistics 24 were used.

## Results

The cohort consisted of 7315 (50.9%) women and 7048 (49.1%) men (Table S1). Among the women, 1326 (18.1%) had long-term WAD and 893 (12.2%) had a PMI, among men the respective numbers were: 925 (13.1%) and 615 (8.7%) (Table S2). In total, two thirds (67%) of occupants with long-term WAD resulted in PMI. Long-term WAD and PMI were among women most common in ages 17–44 years, and among men in ages 30–59 (Table S2).

Further, higher proportions of long-term WAD and PMI occurred among those with previous SA and those already on DP at T_0_. Particularly, a higher proportion of women compared with men already had an ongoing DP at T_0_ (10.7 and 6.5%, respectively) as well as > 90 days of SA in Y_− 1_ (8.1% and 4.9%, respectively) (Table S1). After excluding those with ongoing DP at T_0_, results indicated that 789 (12.1%) women had > 90 days of all-cause SA/DP and 91 (1.4%) had > 90 days SA/DP with WAD diagnosis in Y_+ 2_; while in the group of men, 511 (7.8%) and 80 (1.2%), respectively (Table S3).

**Fig. 1:**
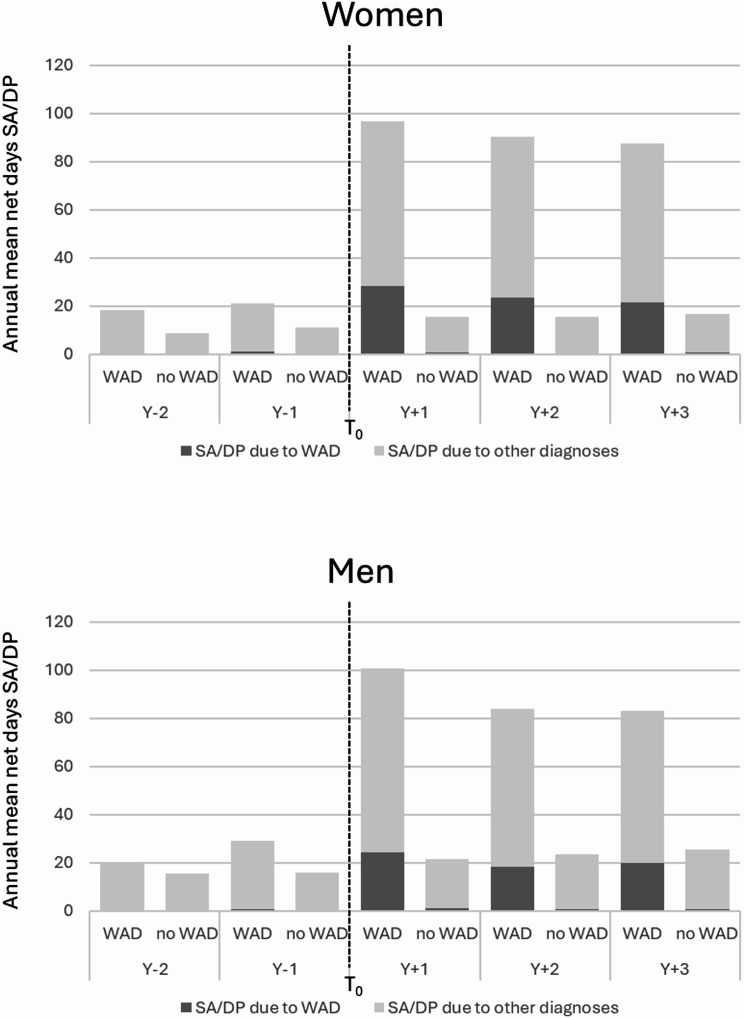
Mean annual number of net days of sickness absence (SA) and disability pension (DP) among individuals not already on SA/DP at the crash date (T_0_), for the period of two years before and three years after T_0_ (Y_-2_ – Y_+ 3_) for all SA/DP and for SA/DP due to whiplash diagnosis, stratified by long-term WAD (LT WAD), for women (*n* = 6155) and men (*n* = 6369), respectively. Loss to follow-up in terms of death and migration was handled in the calculations, why number of individuals can vary by year

In the years following the car crash, the average SA/DP days/year for occupants with long-term WAD increased (Fig. 1). The number of days/year remained at a high level throughout Y_+ 1_-Y_+ 3_. Among persons without long-term WAD, the SA/DP days increased only slightly following the crash.

Regarding the seat design, occupants in older cars (MYI < 1998) had the highest proportions of long-term WAD and PMI (Table S2). Individuals injured in newer cars (MYI ≥ 1998) without WPS had higher proportions of long-term WAD and PMI, compared to those in cars with any kind of WPS (Table S2). However, the proportions varied between specific systems.

**Fig. 2:**
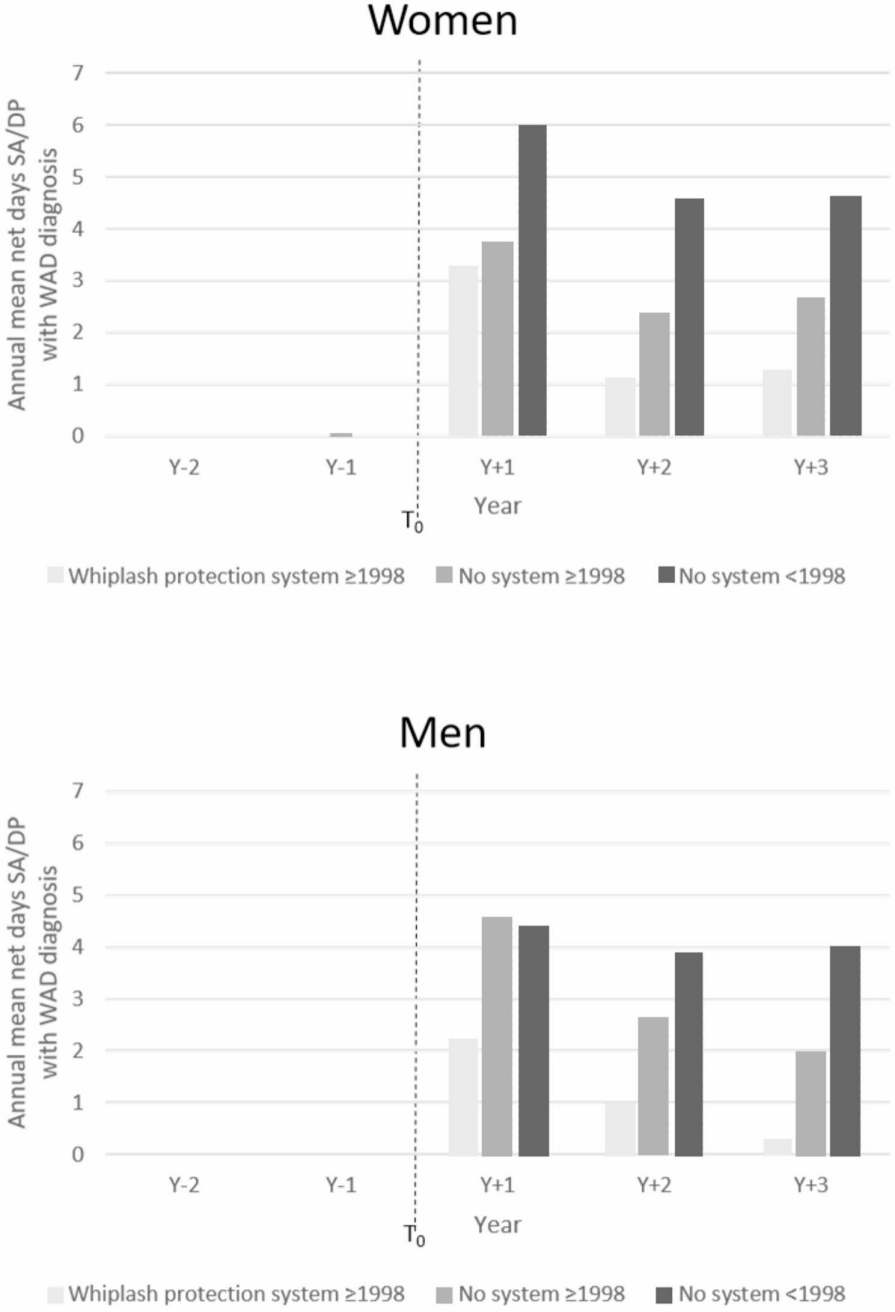
Mean annual number of net days of sickness absence (SA) and disability pension (DP) with WAD diagnosis, among individuals not already with ongoing SA/DP at the car crash date (T_*0*_), for the period of two years before and three years after T_0_ (Y_-2_ – Y_+ 3_), stratified by car seat type, for women (*n* = 6155) and men (*n* = 6369) respectively. Loss to follow-up in terms of death and migration was handled in the calculations, why number of individuals can vary by year

Moreover, occupants injured in older cars (MYI < 1998) had the highest proportion of SA/DP days/year with WAD diagnosis and remained elevated throughout the three years following the crash (Fig. 2). Among women, the difference in SA/DP days in Y_+ 1_ was greater between older and newer cars than between cars with and without a WPS. Among men, the difference was greater between cars with and without WPS. However, Y_+ 2_ and Y_+ 3_, both women and men showed similar mean annual SA/DP patterns, with the highest SA/DP days among users of old cars without WPS, followed by those in newer cars without WPS, and the lowest SA/DP days among users of newer cars with WPS.

In the logistic regression analyses, women injured in older cars (MYI < 1998) were more likely to sustain long-term WAD compared to women in cars with WPS MYI ≥ 1998 (OR: 1.8; CI: 1.5–2.2) (Table 2). Nevertheless, no specific WPS was found to have a statistically significant protective effect compared to STD seats. Men injured in older cars (MYI < 1998) were twice as likely to sustain long-term WAD (OR: 2.2; CI: 1.7–2.8), and men in newer cars (MYI ≥ 1998) without WPS were 60% more likely (OR: 1.6; 1.2-2.0) to sustain long-term WAD compared to men in cars with a WPS. Among the specific systems, PAS_TO and RHR_SAHR had a protective effect, when adjusting for age and car size, compared to STD seats. Further, men injured in older cars and cars without WPS were more likely to have a PMI compared to men in newer cars with WPS (MYI < 1998 OR: 2.3; CI: 1.6–3.3, MYI ≥ 1998 OR: 1.9, CI:1.3–2.7).

**Table 2:** Crude and adjusted odds ratios (OR) with 95% confidence intervals (CI) for long-term WAD and PMI, respectively, in the total 14 363 car occupants with WAD, among women and men, respectively

	Women						Men				
	Crude			Adjusted			Crude			Adjusted	
	OR	95% CI		OR	95% CI		OR	95% CI		OR	95% CI
Long-term WAD^1^											
*Whiplash protection system (WPS): 8 categories*											
STD (Standard seats)	Ref			Ref			Ref			Ref	
PAHR (Pro-Active Head restraints)	1.4	0.3–6.6		1.3	0.3–6.1		0.8	0.2–3.5		0.8	0.2–3.3
PAS (Passive seats)	0.9	0.5–1.6		0.9	0.5–1.6		1.2	0.6–2.4		1.3	0.7–2.5
PAS_TO (Toyota’s passive seats)	0.8	0.6–1.1		0.9	0.7–1.1		**0.4**	**0.2–0.6**		**0.4**	**0.3–0.7**
RHR (Reactive Head Restraints)	1.1	0.8–1.5		1.0	0.7–1.4		0.8	0.5–1.2		0.8	0.5–1.2
RHR_SAHR (SAAB’s RHR)	0.7	0.4–1.2		0.6	0.4–1.1		**0.4**	**0.2–0.9**		**0.4**	**0.2–0.8**
WHIPS (Volvos Reactive seats)	0.9	0.5–1.4		0.8	0.5–1.3		0.9	0.5–1.6		0.9	0.5–1.5
MYI < 1998	**1.5**	**1.4–1.8**		**1.6**	**1.4–1.8**		**1.4**	**1.2–1.7**		**1.4**	**1.2–1.7**
*Whiplash protection system: 3 categories*											
Whiplash protection system MYI ≥ 1998	Ref			Ref			Ref			Ref	
No system MYI ≥ 1998	1.1	0.9–1.4		1.1	0.9–1.4		**1.5**	**1.2-2.0**		**1.6**	**1.2-2.0**
MYI < 1998 (no system available)	**1.8**	**1.5–2.1**		**1.8**	**1.5–2.2**		**2.2**	**1.7–2.8**		**2.2**	**1.7–2.8**
WAD resulting in PMI^2^											
*Whiplash protection system: 8 categories*											
STD (Standard seats)	Ref			Ref			Ref			Ref	
PAHR (Pro-Active Head restraints)	1.1	0.1–8.6		1.6	0.2–12.6		0.6	0.1–4.6		0.9	0.1–6.8
PAS (Passive seats)	0.9	0.4–1.8		1.4	0.6–2.9		0.8	0.3–2.1		1.3	0.5–3.5
PAS_TO (Toyotas passive seats)	0.7	0.5-1.0		0.9	0.6–1.3		**0.3**	**0.2–0.6**		**0.4**	**0.2–0.8**
RHR (Reactive Head Restraints	1.1	0.7–1.7		1.5	1.0-2.3		0.5	0.3-1.0		0.7	0.4–1.4
RHR_SAHR (SAABs RHR)	0.9	0.5–1.6		1.0	0.5–1.9		**0.2**	**0.1–0.7**		**0.2**	**0.1–0.8**
WHIPS (Volvos Reactive seats)	0.6	0.3–1.1		0.7	0.3–1.5		0.4	0.2–1.1		0.5	0.2–1.2
MYI < 1998	**1.9**	**1.6–2.2**		**1.4**	**1.2–1.7**		**1.5**	**1.2–1.8**		**1.2**	**1.0-1.5**
*Whiplash protection system: 3 categories*											
Whiplash protection system MYI ≥ 1998	Ref			Ref			Ref			Ref	
No system MYI ≥ 1998	1.2	1.0-1.6		1.0	0.8–1.3		**2.4**	**1.6–3.4**		**1.9**	**1.3–2.7**
MYI < 1998 (no system available)	**2.3**	**1.9–2.9**		**1.4**	**1.1–1.8**		**3.5**	**2.5-5.0**		**2.3**	**1.6–3.3**

*OR* odds ratio, *CI* confidence interval, *WAD* whiplash-associated disorders, *PMI* permanent medical impairment, *MYI* car model year of introduction

^1^Adjustments made for Age and Car size

^2^Adjustments made for Age, Car size, and Year of injury

Values in bold indicate statistically significant results.

Men injured in cars MYI < 1998 were also more likely to have > 90 days all-cause SA/DP two years after the crash (Y_+ 2_), compared with men in newer cars with WPS (OR: 1.8; CI: 1.2–2.6) (Table 3). Additionally, around a third of individuals with long-term WAD or PMI had > 90 days all-cause SA/DP in Y_+ 2_ (Table S3). Among both those without long-term WAD or without PMI, 8.5% of women and 4.6% of men had > 90 days all-cause SA/DP in Y_+ 2_. To have > 90 days all-cause SA/DP in Y_+ 2_ was also more common among women, among those with previous SA, as well as among occupants in older cars (MYI < 1998).

**Table 3:** Crude and adjusted odds ratios (OR) with 95% confidence intervals (CI) for > 90 days of all-cause SA/DP and > 90 days of SA/DP with whiplash diagnosis, respectively, in year two after the crash (Y_+ 2_) among car occupants with WAD and not on already ongoing DP at the crash date (*n* = 13 089). For women and men, respectively

	Women						Men				
	Crude			Adjusted^1^			Crude			Adjusted^1^	
	OR	95% CI		OR	95% CI		OR	95% CI		OR	95% CI
All-cause SA/DP											
*Whiplash protection system (WPS) 8 categories*											
STD (Standard seats)	Ref			Ref			Ref			Ref	
PAHR (Pro-Active Head restraints)	**5.1**	**1.2–21.6**		4.5	0.8–25.4		0.8	0.1–6.3		1.3	0.2–10.3
PAS (Passive seats)	1.1	0.5–2.1		1.4	0.7–2.9		1.1	0.5–2.7		1.4	0.5–3.7
PAS_TO (Toyotas passive seats)	0.8	0.6–1.1		0.9	0.6–1.2		**0.4**	**0.2–0.8**		0.6	0.3–1.1
RHR (Reactive Head Restraints)	0.8	0.5–1.3		1.0	0.6–1.6		0.6	0.3–1.1		0.8	0.4–1.6
RHR_SAHR (SAABs RHR)	1.1	0.6–1.9		1.1	0.6–1.9		0.4	0.1-1.0		0.5	0.2–1.4
WHIPS (Volvos Reactive seats)	0.5	0.2–1.1		0.6	0.3–1.4		0.5	0.2–1.3		0.6	0.2–1.6
MYI < 1998	**1.4**	**1.2–1.7**		1.2	1.0-1.4		**1.5**	**1.2–1.9**		1.2	1.0-1.5
*Whiplash protection system 3 categories*											
Whiplash protection system MYI ≥ 1998	Ref			Ref			Ref			Ref	
No system MYI ≥ 1998	1.2	0.9–1.5		1.1	0.8–1.4		**1.9**	**1.3–2.7**		1.4	1.0-2.2
MYI < 1998 (no system available)	**1.7**	**1.4–2.1**		1.3	1.0-1.7		**2.8**	**2.0-3.9**		**1.8**	**1.2–2.6**
SA/DP with WAD diagnosis											
*Whiplash protection system 3 categories*											
Whiplash protection system MYI ≥ 1998	Ref			Ref			Ref			Ref	
No system MYI ≥ 1998	2.2	0.9–5.3		1.6	0.6-4.0		**6.5**	**1.5–27.4**		3.3	0.7–14.2
MYI < 1998 (no system available)	**3.8**	**1.7–8.9**		2.0	0.8–5.1		**7.9**	**1.9–32.3**		3.0	0.7–13.1

*OR* odds ratio, *CI* confidence interval, *SA* sickness absence, *DP* disability pension, *WAD* whiplash-associated disorders, *MYI* car model year of introduction

^1^Adjusted for Age, Education level, Country of birth, Civil status, Previous SA, Year of injury and Car size

Values in bold indicate statistically significant results.

## Discussion

In this large longitudinal cohort study of individuals of working age reporting WAD injuries sustained as front seat car occupants in rear-end crashes, 18% of the women and 13% of the men experienced long-term WAD (defined as receiving insurance compensation for four weeks or more and based on medical assessment), and about two thirds of these cases resulted in PMI. Both long-term WAD and PMI occurred in a higher proportion among the women than among the men. The SA/DP days/year increased substantially among those with long-term WAD and remained on a high level during the three years following the crash. Women in cars with WPS were less likely to have long-term WAD and PMI compared to women in older cars but not compared to women in newer cars without WPS. Men in cars equipped with WPS were less likely to have long-term WAD and PMI, compared with men in both newer and older cars not equipped with such systems, and less likely to have high SA/DP in Y_+ 2_, compared to those in older cars.

### Mean number of SA/DP days per Year

The SA/DP days/year due to WAD diagnosis were almost exclusively found among those with long-term WAD; this group had substantial work incapacity during several years following the crash. This finding supports previous studies, reporting remaining disability for several years following the crash [9, 10, 23].This work incapacity was not only with WAD diagnoses, all-cause SA/DP days (with any diagnosis) also increased after the crash, in line with our previous findings [12, 13]. This suggests a distinct difference regarding impact on the lives of those injured, in terms of future work incapacity between individuals with WAD with different symptom duration. That both SA/DP with WAD diagnosis and all-cause SA/DP increased after the crash among individuals with a high number of SA days before the crash (Y_− 1_) may support the findings of three other studies that those with poor health already before the crash are more vulnerable and thus at higher risk of WAD [11, 24, 25]. They may also have pre-existing conditions that are exacerbated due to the crash, e.g., neck pain is a common condition in the general population [26]. More studies regarding this are needed. It is important to note that SA/DP reflect not only work incapacity due to the injury but also factors such as work conditions, social insurance rules, and prior health status, and should therefore not be interpreted as a causal consequence of WAD alone [27].

### Associations with whiplash protection system

The results of this study are in line with previous findings indicating that the risk of long-term WAD and of WAD resulting in PMI are lower for occupants in newer cars [3, 28]. Whiplash protection systems (WPS) are more common in modern cars and Kullgren et al. (2013) found that injured occupants had a 40% lower risk of WAD resulting in PMI for cars equipped with a WPS compared with cars without WPS of model year ≥ 1998 [3]. The effect was higher for men than for women (50% compared to 30%). The results of the present study support this pattern, indicating that WPS were associated with lower risk but that this association was notably weaker for women than for men, regarding long-term WAD, PMI, and SA/DP. The findings of this study and the one by Kullgren et al. (2013) showing that current WPS seem to be more beneficial to men than to women are especially alarming due to the fact that women’s risk of WAD is up to three times higher that of men [29]. Furthermore, in line with our results, women have also been reported to have a higher risk of WAD resulting in PMI [5]. Different dynamic responses between women and men in rear-end impacts and differing interactions with the seat back depending on the size of occupants have been reported [29]. Given that WPS are assessed using the 50th percentile male dummy [18, 30], this may explain the differences between women and men seen in this and other studies regarding outcomes of varying seat concepts. Indeed, a study testing head/neck responses to rear-impact crashes using a prototype of a 50th percentile female dummy, demonstrated that the male dummy does not accurately represent the average female’s response to dynamic seat performance and associated protective effects of WPS [31].

### Strengths and limitations

Strengths include the use of a large cohort, which allowed stratified sub-group analyses and to control for potential confounders, such as crash year, car size, and sociodemographics. Moreover, a sex stratification was possible due to the large cohort, which enhanced the possibility of establishing sex differences among different WPS in real-world data. Further, the use of high-quality data from nationwide registers meant that individuals could be identified as injured from two sources for a more complete cohort, which could be followed regarding SA and DP. Thereby, those already on DP at the crash date could be excluded for some analyses, and previous history of SA could be taken into consideration. Another strength was the use of net days instead of gross days of SA and DP, to take grade of SA/DP into consideration. We could also exclude those who during follow up were not at risk for SA and DP due to migration and death, which may lead to a slight underestimation of SA/DP if their health trajectories differed from those who remained in the cohort. Nevertheless, only a small number of participants died or emigrated during follow‑up. 

The large cohort allowed for several important sub-group analyses. Nevertheless, it was a limitation that some of the sub-groups were too small to allow meaningful analyses, e.g., regarding the associations between specific WPS and SA/DP with WAD diagnosis.

Encouragingly, we found that few occupants in cars equipped with WPS had > 90 days of SA/DP with WAD diagnoses when followed up in Y_+ 2_. However, the small number in some cells meant that the CIs were wide and results should be considered with caution.

Because the study sample includes only individuals who sustained an injury, selection bias may occur - factors unrelated to age or car size that influence the likelihood of injury can become spuriously associated with whiplash protection systems. If these same factors also relate to WAD, the resulting bias could distort the observed association in either direction. Another limitation is that we did not have information on SA spells ≤ 14 days, which means a slight underestimation of the number of SA days in those shorter spells. However, we catch the more serious SA and the very short SA spells stands for a small number of all SA days.

Unfortunately, we did not have access to detailed information regarding the severity of whiplash injuries or crash-related factors like vehicle speed and size. This limits the depth of analysis and the ability to draw more precise comparisons. Another limitation is that we did not have information on, e.g., stature or body weight, factors that have been found to influence the risk of WAD [32].

 The present study was based on the insurance policyholders’ claim reports to their insurance company (Folksam). The reliance on a single insurer does not introduce under‑coverage within the cohort, as all included individuals were Folksam policyholders who reported their crashes. Such reports are mandatory in order to receive payment for expenses and occupant injuries. Therefore, occupants with minor injuries are also included. This makes the data more accurate and prevents overestimation of long-term WAD.

As some outcomes, especially among women, were in a range where odds ratios can somewhat overestimate relative risks, the reported associations should be interpreted with this consideration in mind. Nevertheless, given the modest effect sizes and generally low baseline risks, any inflation is likely to be small and does not materially affect the study’s conclusions. Furthermore, it was possible to follow long-term WAD as we defined as those injuries with symptoms for ≥four weeks. Thereby, over-reporting of WAD could be avoided, and those with no or very short-term symptoms could be identified. However, proportions presented here are thusly only related to those reporting WAD. We probably underestimate associations between types of whiplash protection system and long-term WAD, PMI, and SA/DP concerning numbers and rates not injured due to the use of protection systems. Nevertheless, insurance claims data includes higher proportions of WAD than, e.g., data gathered from hospital emergency wards or police reports.

## Conclusions

We found that among front‑seat occupants who sustained a WAD injury in a rear‑end crash, 18% of women and 13% of men developed long-term WAD (symptoms lasting over four weeks). In this group, both the all-cause mean SA/DP days and the mean SA/DP days with WAD diagnosis increased substantially and remained on a high level during the three years following the crash. Whiplash protection systems were associated with less long-term WAD, WAD resulting in PMI, and SA/DP, however, to a larger extent among men than women. This clearly supports the need for preventive interventions for reducing the risk of WAD among women. 

## Supplementary Information

Supplementary Material 1.

## Data Availability

The data used in the study cannot be made public. Such data can only be made available, after legal review, to researchers who meet the criteria to access such sensitive and confidential data, according to the General Data Protection Regulation, the Swedish Data Protection Act, the Swedish Ethical Review Act and the Swedish Public Access to Information and Secrecy Act. Readers may contact Associate Professor Emilie Friberg (emilie.friberg@ki.se) regarding these data.

## Abbreviations

CI: Confidence Intervals
DP: Disability Pension
ICD: International Classification of Diseases
LISA: Longitudinal Integration Database for Health Insurance and Labour Market Studies
MiDAS: MicroData for Analysis of the Social Insurance database
MYI: Model year of introduction (MYI)
NPR: National patient register
OR: Odds ratio
PAHR: Pro-Active Head restraints
PAS: Passive seats
PAS_TO: Toyota’s passive seats
PMI: Permanent medical Impairment
RHR: Reactive Head Restraints
RHR_SAHR: SAAB’s Reactive Head Restraints SA
STD: Standard seats
WAD: Whiplash-associated disorders
WHIPS: Volvos Reactive seats
WPS: Whiplash protectives systems
