# Supplementary material for: Long-term whiplash-associated disorders, sickness absence, and disability pension following rear-end car crashes and associations with whiplash protection systems: a longitudinal cohort study
Source: BMC Public Health. 2026 Apr 20;26:1279. doi: 10.1186/s12889-026-27404-2 (PMC13094100; doi:10.1186/s12889-026-27404-2)
Supplement: Supplementary file 1 — Supplementary Material 1. [file 12889_2026_27404_MOESM1_ESM.docx]

**Appendix**

Table A1. Characteristics of the studied cohort of passenger car occupants with WAD injury (N=14,363), for women and men, respectively.

|  | **Women** | | **Men** | |
| --- | --- | --- | --- | --- |
| **Sociodemographic factors** | N=7315 | % | N=7048 | % |
| *Age groups (year)* |  |  |  |  |
| 17-29 | 1696 | 23.2 | 1914 | 27.2 |
| 30-44 | 2869 | 39.2 | 2470 | 35.0 |
| 45-59 | 2396 | 32.8 | 2303 | 32.7 |
| 60-62 | 354 | 4.8 | 361 | 5.1 |
| *Country of birth* |  |  |  |  |
| Sweden | 6270 | 85.7 | 5378 | 76.3 |
| Rest of Europe | 543 | 7.4 | 684 | 9.7 |
| Rest of the world | 502 | 6.9 | 986 | 14.0 |
| *Level of education* |  |  |  |  |
| Elementary | 901 | 12.3 | 1451 | 20.6 |
| High school | 4017 | 54.9 | 3870 | 54.9 |
| College/University | 2397 | 32.8 | 1727 | 24.5 |
| *Married* |  |  |  |  |
| Yes | 2799 | 38.3 | 2812 | 39.9 |
| No | 4516 | 61.7 | 4236 | 60.1 |
| *SA/DP status at T_0_* |  |  |  |  |
| No SA/DP | 6155 | 84.1 | 6369 | 90.4 |
| Already ongoing SA | 379 | 5.2 | 219 | 3.1 |
| Already ongoing DP | 781 | 10.7 | 460 | 6.5 |
| *Previous SA (>90 days in the year before T_0_)* |  |  |  |  |
| Yes | 596 | 8.1 | 343 | 4.9 |
| No | 6719 | 91.9 | 6705 | 95.1 |
| **Car-related factors** |  |  |  |  |
| *Seat design* |  |  |  |  |
| Whiplash protection system MYI≥1998 | 1293 | 17.7 | 1082 | 15.4 |
| No system MYI≥1998 | 2506 | 34.3 | 2070 | 29.4 |
| MYI<1998 (no system available) | 3516 | 48.1 | 3896 | 55.3 |
| *Car model year of introduction* |  |  |  |  |
| 2009-2013 | 115 | 1.6 | 94 | 1.3 |
| 2004-2008 | 697 | 9.5 | 519 | 7.4 |
| 1999-2003 | 1251 | 17.1 | 884 | 12.5 |
| 1994-1998 | 1803 | 24.6 | 1680 | 23.8 |
| 1993 or older | 3449 | 47.1 | 3871 | 54.9 |
| *Car size* |  |  |  |  |
| Superminis | 1512 | 20.7 | 765 | 10.9 |
| Small family cars | 2067 | 28.3 | 1643 | 23.3 |
| Large family cars | 1641 | 22.4 | 1949 | 27.7 |
| Executive cars | 1347 | 18.4 | 1870 | 26.5 |
| Minibuses | 244 | 3.3 | 221 | 3.1 |
| SUV | 206 | 2.8 | 159 | 2.3 |
| Missing information | 298 | 4.1 | 441 | 6.3 |
| *Year of crash* |  |  |  |  |
| 2009-2013 | 2358 | 32.2 | 1911 | 27.1 |
| 2005-2008 | 2037 | 27.8 | 2007 | 28.5 |
| 2001-2004 | 2920 | 39.9 | 3130 | 44.4 |

SA: Sickness absence, DP: Disability pension, T_0_: crash date, SUV: sport utility vehicle, MYI: Model year of introduction

Table A2. Numbers and proportions (%) of individuals (n=14 363) with long-term WAD and WAD resulting in PMI, stratified by sociodemographics and car-related factors. For women and men, respectively.

|  |  | **Women** | | | | | | |  | **Men** | | | | | | |
| --- | --- | --- | --- | --- | --- | --- | --- | --- | --- | --- | --- | --- | --- | --- | --- | --- |
|  |  |  |  | Long-term WAD | |  | WAD resulting in PMI | |  |  |  | Long-term WAD | |  | WAD resulting in PMI | |
|  |  | n |  | n | % |  | n | % |  | n |  | n | % |  | n | % |
| Total |  | 7315 |  | 1326 | 18.1 |  | 893 | 12.2 |  | 7048 |  | 925 | 13.1 |  | 615 | 8.7 |
| Age | 17-29 | 1696 |  | 314 | 18.5 |  | 226 | 13.3 |  | 1914 |  | 203 | 10.6 |  | 139 | 7.3 |
|  | 30-44 | 2869 |  | 536 | 18.7 |  | 366 | 12.8 |  | 2470 |  | 336 | 13.6 |  | 220 | 8.9 |
|  | 45-59 | 2396 |  | 415 | 17.3 |  | 266 | 11.1 |  | 2303 |  | 348 | 15.1 |  | 232 | 10.1 |
|  | 60-62 | 354 |  | 61 | 17.2 |  | 35 | 9.9 |  | 361 |  | 38 | 10.5 |  | 24 | 6.6 |
| Previous SA | >90 days in Y_-1_ | 596 |  | 159 | 26.7 |  | 108 | 18.1 |  | 343 |  | 81 | 23.6 |  | 56 | 16.3 |
|  | ≤90 days in Y_-1_ | 6719 |  | 1167 | 17.4 |  | 785 | 11.7 |  | 6705 |  | 844 | 12.6 |  | 559 | 8.3 |
| Already ongoing DP at T_0_ | Yes | 781 |  | 228 | 29.2 |  | 135 | 17.3 |  | 460 |  | 117 | 25.4 |  | 76 | 16.5 |
|  | No | 6534 |  | 1098 | 16.8 |  | 758 | 11.6 |  | 6588 |  | 808 | 12.3 |  | 539 | 8.2 |
| Whiplash protection system  (3 categories) | Whiplash protection system MYI≥1998 | 1293 |  | 176 | 13.6 |  | 98 | 7.6 |  | 1082 |  | 84 | 7.8 |  | 36 | 3.3 |
|  | No system≥1998 | 2506 |  | 383 | 15.3 |  | 232 | 9.3 |  | 2070 |  | 238 | 11.5 |  | 156 | 7.5 |
|  | MYI<1998 (no system available) | 3516 |  | 767 | 21.8 |  | 563 | 16.0 |  | 3896 |  | 603 | 15.5 |  | 423 | 10.9 |
| Whiplash protection system  (8 categories) | PAHR (Pro-Active Head restraints) | 10 |  | <10 | 20.0 |  | <10 | 10.0 |  | 21 |  | <10 | 9.5 |  | <10 | 4.8 |
|  | PAS (Passive seats) | 100 |  | 14 | 14.0 |  | <10 | 8.0 |  | 80 |  | 11 | 13.8 |  | <10 | 6.3 |
|  | PAS_TO (Toyota’s passive seats) | 609 |  | 79 | 13.0 |  | 41 | 6.7 |  | 390 |  | 19 | 4.9 |  | 10 | 2.6 |
|  | RHR (Reactive Head Restraints) | 279 |  | 45 | 16.1 |  | 28 | 10.0 |  | 281 |  | 27 | 9.6 |  | 12 | 4.3 |
|  | RHR_SAHR (SAAB’s RHR) | 145 |  | 16 | 11.0 |  | 12 | 8.3 |  | 163 |  | <10 | 5.5 |  | <10 | 1.8 |
|  | WHIPS (Volvo’s Reactive seats) | 150 |  | 20 | 13.3 |  | <10 | 5.3 |  | 147 |  | 16 | 10.9 |  | <10 | 3.4 |
|  | STD (Standard seats) | 2506 |  | 383 | 15.3 |  | 232 | 9.3 |  | 2070 |  | 238 | 11.5 |  | 156 | 7.5 |
|  | MYI<1998 | 3516 |  | 767 | 21.8 |  | 563 | 16.0 |  | 3896 |  | 603 | 15.5 |  | 423 | 10.9 |
| Car size | Superminis | 1512 |  | 237 | 15.7 |  | 144 | 9.5 |  | 765 |  | 87 | 11.4 |  | 52 | 6.8 |
|  | Small family cars | 2067 |  | 388 | 18.8 |  | 269 | 13.0 |  | 1643 |  | 199 | 12.1 |  | 134 | 8.2 |
|  | Large family cars | 1641 |  | 332 | 20.2 |  | 226 | 13.8 |  | 1949 |  | 284 | 14.6 |  | 189 | 9.7 |
|  | Executive cars | 1347 |  | 253 | 18.8 |  | 181 | 13.4 |  | 1870 |  | 261 | 14.0 |  | 180 | 9.6 |
|  | Minibuses | 244 |  | 46 | 18.9 |  | 33 | 13.5 |  | 221 |  | 22 | 10.0 |  | 10 | 4.5 |
|  | SUVs | 206 |  | 24 | 11.7 |  | 11 | 5.3 |  | 159 |  | <10 | 5.7 |  | <10 | 3.8 |
|  | Missing information | 298 |  | 46 | 15.4 |  | 29 | 9.7 |  | 441 |  | 63 | 14.3 |  | 44 | 10.0 |
| Year of the crash | 2009-2013 | 2358 |  | 290 | 12.3 |  | 154 | 6.5 |  | 1911 |  | 172 | 9.0 |  | 84 | 4.4 |
|  | 2005-2008 | 2037 |  | 313 | 15.4 |  | 192 | 9.4 |  | 2007 |  | 212 | 10.6 |  | 136 | 6.8 |
|  | 2001-2004 | 2920 |  | 723 | 24.8 |  | 547 | 18.7 |  | 3130 |  | 541 | 17.3 |  | 395 | 12.6 |

WAD: Whiplash-associated disorders, PMI: permanent medical impairment, SA: sickness absence, DP: disability pension, T_0_: crash date, SUV: sport utility vehicle.

For ethical reasons and in accordance with suppression rules, counts less than 10 are indicated as <10.

Table A3. Numbers and proportions (%) with >90 days of all-cause SA/DP and >90 days of SA/DP with WAD diagnosis in Y_+2_, among individuals at risk of SA/DP in terms of not already ongoing DP at crash, not died or migrated (n=13,089), stratified by sociodemographics, injury- and car-related factors. For women and men, respectively

|  |  | **Women** | | | | | | |  | **Men** | | | | | | |
| --- | --- | --- | --- | --- | --- | --- | --- | --- | --- | --- | --- | --- | --- | --- | --- | --- |
|  |  |  |  | >90 days all-cause SA/DP | | >90 days SA/DP with WAD diagnosis | |  |  | |  | >90 days all-cause SA/DP | | >90 days SA/DP with WAD diagnosis | |  |
|  |  | n |  | n | % | n | % |  | n | |  | n | % | n | % |  |
| Total |  | 6519 |  | 789 | 12.1 | 91 | 1.4 |  | 6570 | |  | 511 | 7.8 | 80 | 1.2 |  |
| Age | 17-29 | 1678 |  | 116 | 6.9 | 14 | 0.8 |  | 1895 | |  | 84 | 4.4 | 14 | 0.7 |  |
|  | 30-44 | 2671 |  | 368 | 13.8 | 51 | 1.9 |  | 2377 | |  | 172 | 7.2 | 33 | 1.4 |  |
|  | 45-59 | 1915 |  | 259 | 13.5 | 20 | 1.0 |  | 2038 | |  | 223 | 10.9 | 32 | 1.6 |  |
|  | 60-62 | 255 |  | 46 | 18.0 | <10 | 2.4 |  | 260 | |  | 32 | 12.3 | <10 | 0.4 |  |
| Educational level | Elementary | 731 |  | 124 | 17.0 | 12 | 1.6 |  | 1285 | |  | 122 | 9.5 | 15 | 1.2 |  |
|  | High school | 3560 |  | 469 | 13.2 | 65 | 1.8 |  | 3621 | |  | 294 | 8.1 | 49 | 1.4 |  |
|  | College/University | 2228 |  | 196 | 8.8 | 14 | 0.6 |  | 1664 | |  | 95 | 5.7 | 16 | 1.0 |  |
| Country of birth | Sweden | 5624 |  | 634 | 11.3 | 65 | 1.2 |  | 5057 | |  | 308 | 6.1 | 43 | 0.9 |  |
|  | Europe other than Sweden | 443 |  | 75 | 16.9 | 15 | 3.4 |  | 612 | |  | 93 | 15.2 | 17 | 2.8 |  |
|  | Rest of the world | 452 |  | 80 | 17.7 | 11 | 2.4 |  | 901 | |  | 110 | 12.2 | 20 | 2.2 |  |
| Civil status | Married | 2439 |  | 317 | 13.0 | 35 | 1.4 |  | 2571 | |  | 258 | 10.0 | 49 | 1.9 |  |
|  | Not married | 4080 |  | 472 | 11.6 | 56 | 1.4 |  | 3999 | |  | 253 | 6.3 | 31 | 0.8 |  |
| Previous SA | >90 days in Y_-1_ | 409 |  | 217 | 53.1 | 15 | 3.7 |  | 245 | |  | 122 | 49.8 | 13 | 5.3 |  |
|  | ≤90 days in Y_-1_ | 6110 |  | 572 | 9.4 | 76 | 1.2 |  | 6325 | |  | 389 | 6.2 | 67 | 1.1 |  |
| Year of the crash | 2009-2013 | 2173 |  | 193 | 8.9 | 13 | 0.6 |  | 1816 | |  | 74 | 4.1 | <10 | 0.2 |  |
|  | 2005-2008 | 1753 |  | 167 | 9.5 | 18 | 1.0 |  | 1855 | |  | 104 | 5.6 | 18 | 1.0 |  |
|  | 2001-2004 | 2593 |  | 429 | 16.5 | 60 | 2.3 |  | 2899 | |  | 333 | 11.5 | 59 | 2.0 |  |
| Long-term WAD | WAD | 1097 |  | 327 | 29.8 | 78 | 7.1 |  | 807 | |  | 248 | 30.7 | 71 | 8.8 |  |
|  | No long-term WAD | 5422 |  | 462 | 8.5 | 13 | 0.2 |  | 5763 | |  | 263 | 4.6 | <10 | 0.2 |  |
| WAD resulting in PMI | PMI ≥1% | 758 |  | 253 | 33.4 | 71 | 9.4 |  | 538 | |  | 195 | 36.2 | 59 | 11.0 |  |
|  | No PMI | 5761 |  | 536 | 9.3 | 20 | 0.3 |  | 6032 | |  | 316 | 5.2 | 21 | 0.3 |  |
| Whiplash protection system (3 categories) | Whiplash protection system MYI≥1998 | 1171 |  | 106 | 9.1 | <10 | 0.5 |  | 1034 | |  | 38 | 3.7 | <10 | 0.2 |  |
|  | No system MYI≥1998 | 2251 |  | 236 | 10.5 | 25 | 1.1 |  | 1938 | |  | 128 | 6.6 | 24 | 1.2 |  |
|  | MYI<1998 (no system available) | 3097 |  | 447 | 14.4 | 60 | 1.9 |  | 3598 | |  | 345 | 9.6 | 54 | 1.5 |  |
| (cont. next page)  Whiplash protection system (8 categories) | PAHR (Pro-Active Head restraints) | 8 |  | 3 | 37.5 | 0 | 0.0 |  | 18 | |  | 1 | 5.6 | 0 | 0.0 |  |
|  | PAS (Passive seats) | 90 |  | 10 | 11.1 | <10 | 0.0 |  | 80 | |  | <10 | 7.5 | <10 | 1.3 |  |
|  | PAS_TO (Toyotas passive seats) | 547 |  | 47 | 8.6 | <10 | 0.5 |  | 374 | |  | 11 | 2.9 | <10 | 0.3 |  |
|  | RHR (Reactive Head Restraints) | 255 |  | 23 | 9.0 | <10 | 0.8 |  | 266 | |  | 11 | 4.1 | <10 | 0.0 |  |
|  | RHR_SAHR (SAABs RHR) | 130 |  | 15 | 11.5 | <10 | 0.8 |  | 156 | |  | <10 | 2.6 | <10 | 0.0 |  |
|  | WHIPS (Volvos Reactive seats) | 141 |  | <10 | 5.7 | <10 | 0.0 |  | 140 | |  | <10 | 3.6 | <10 | 0.0 |  |
|  | STD (Standard seats) | 2251 |  | 236 | 10.5 | 25 | 1.1 |  | 1938 | |  | 128 | 6.6 | 24 | 1.2 |  |
|  | MYI<1998 | 3097 |  | 447 | 14.4 | 60 | 1.9 |  | 3598 | |  | 345 | 9.6 | 54 | 1.5 |  |
| Car size | Superminis | 1357 |  | 147 | 10.8 | 13 | 1.0 |  | 735 | |  | 47 | 6.4 | <10 | 1.2 |  |
|  | Small family cars | 1856 |  | 231 | 12.4 | 34 | 1.8 |  | 1536 | |  | 110 | 7.2 | 16 | 1.0 |  |
|  | Large family cars | 1456 |  | 169 | 11.6 | 21 | 1.4 |  | 1803 | |  | 159 | 8.8 | 28 | 1.6 |  |
|  | Executive cars | 1188 |  | 152 | 12.8 | 14 | 1.2 |  | 1742 | |  | 143 | 8.2 | 20 | 1.1 |  |
|  | Minibuses | 215 |  | 30 | 14.0 | <10 | 0.5 |  | 200 | |  | 14 | 7.0 | <10 | 1.0 |  |
|  | SUVs | 181 |  | 24 | 13.3 | <10 | 1.7 |  | 147 | |  | <10 | 4.8 | <10 | 0.7 |  |
|  | Missing information | 266 |  | 36 | 13.5 | <10 | 1.9 |  | 407 | |  | 31 | 7.6 | <10 | 1.0 |  |

SA: sickness absence, DP: disability pension, Y_-1_: The year before the crash date, WAD: Whiplash-associated disorders, PMI: permanent medical impairment, MYI: car model year of introduction, SUV: sport utility vehicle

For ethical reasons and in accordance with suppression rules, counts less than 10 are indicated as <10.
